# Publisher Correction: Laser-excited elastic guided waves reveal the complex mechanics of nanoporous silicon

**DOI:** 10.1038/s41467-021-24354-8

**Published:** 2021-06-22

**Authors:** Marc Thelen, Nicolas Bochud, Manuel Brinker, Claire Prada, Patrick Huber

**Affiliations:** 1 Hamburg University of Technology, Institute of Materials and X-Ray Physics, Hamburg, Germany; 2 MSME, CNRS UMR 8208, Univ Paris Est Creteil, Univ Gustave Eiffel, Creteil, France; 3 Institut Langevin, ESPCI Paris, Université Paris Sciences et Lettres, CNRS, Paris, France; 4 Deutsches Elektronen-Synchrotron DESY, Centre for X-Ray and Nano Science CXNS, Hamburg, Germany; 5 Hamburg University, Centre for Hybrid Nanostructures CHyN, Hamburg, Germany

**Keywords:** Nanoscale materials, Acoustics, Characterization and analytical techniques

Correction to: *Nature Communications* 10.1038/s41467-021-23398-0 10.1038/s41467-021-23398-0, published online 14 June 2021

The original version of this article contained an error in the author affiliations.

Patrick Huber was incorrectly associated with MSME, CNRS UMR 8208, Univ Paris Est Creteil, Univ Gustave Eiffel, Creteil, France.

This has now been corrected in both the PDF and HTML versions of the article.

