# Supplementary material for: Laser-excited elastic guided waves reveal the complex mechanics of nanoporous silicon
Source: Nat Commun. 2021 Jun 14;12:3597. doi: 10.1038/s41467-021-23398-0 (PMC8203614; doi:10.1038/s41467-021-23398-0)
Supplement: Supplementary file 1 — Supplementary Information [file 41467_2021_23398_MOESM1_ESM.pdf]

Supplementary Information
Laser-Excited Elastic Guided Waves Reveal the Complex Mechanics of Nanoporous Silicon

Marc Thelen,[1,*] Nicolas Bochud,[2,†] Manuel Brinker,[1,‡] Claire Prada,[3,§] and Patrick Huber[1,4,5,¶]

[1] *Hamburg University of Technology, Institute of Materials and X-Ray Physics, 21073 Hamburg, Germany.*
[2] *MSME, CNRS UMR 8208, Univ Paris Est Creteil, Univ Gustave Eiffel, F-94010 Creteil, France.*
[3] *Institut Langevin, ESPCI Paris, Université Paris Sciences et Lettres, CNRS, 75005 Paris, France.*
[4] *Deutsches Elektronen-Synchrotron DESY, Centre for X-Ray and Nano Science CXNS, 22603 Hamburg, Germany.*
[5] *Hamburg University, Centre for Hybrid Nanostructures CHyN, 22607 Hamburg, Germany.*

(Dated: April 9, 2021)

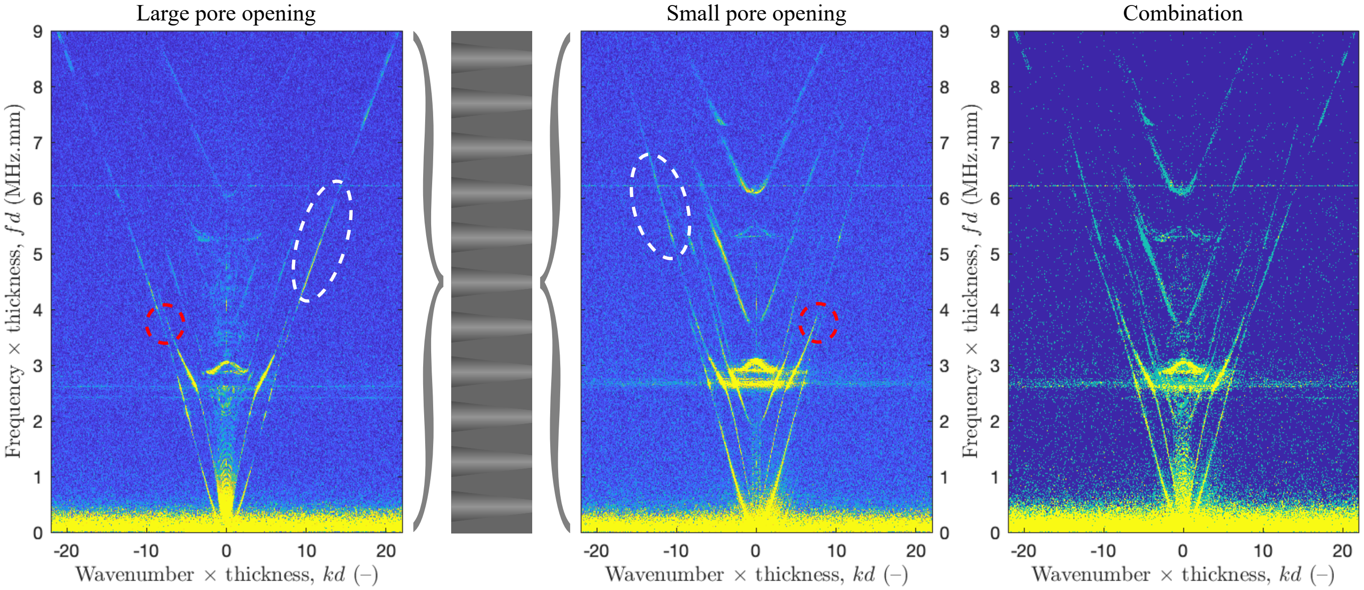

Supplementary Fig. 1: **Side dependent Rayleigh wave velocities.** Dispersion spectra derived from LUS measurements performed on both sides of a pSi membrane with similar characteristics to that presented in the manuscript. Zero-order symmetric (qS_0) and antisymmetric (qA_0) modes do not converge to the same Rayleigh wave velocity due to the break in symmetry at around $kd \approx 8$ (red circles) caused by the cone-shaped pores. This results in two different Rayleigh (surface) waves, measured with different intensities on each side (white circles) of the sample. Larger pore opening and hence higher porosity on one side of the sample (a) leads to a lower Rayleigh velocity compared to that on the side with smaller pore opening (b). The combination of both dispersion spectra (c) shows that, in contrast to the lowest order modes, the higher order modes are not impacted by the cone-shaped pores.

[*] These two authors contributed equally; marc.thelen@tuhh.de
[†] These two authors contributed equally; nicolas.bochud@u-pec.fr
[‡] manuel.brinker@tuhh.de
[§] claire.prada@espci.psl.eu
[¶] patrick.huber@tuhh.de
https://huberlab.wp.tuhh.de

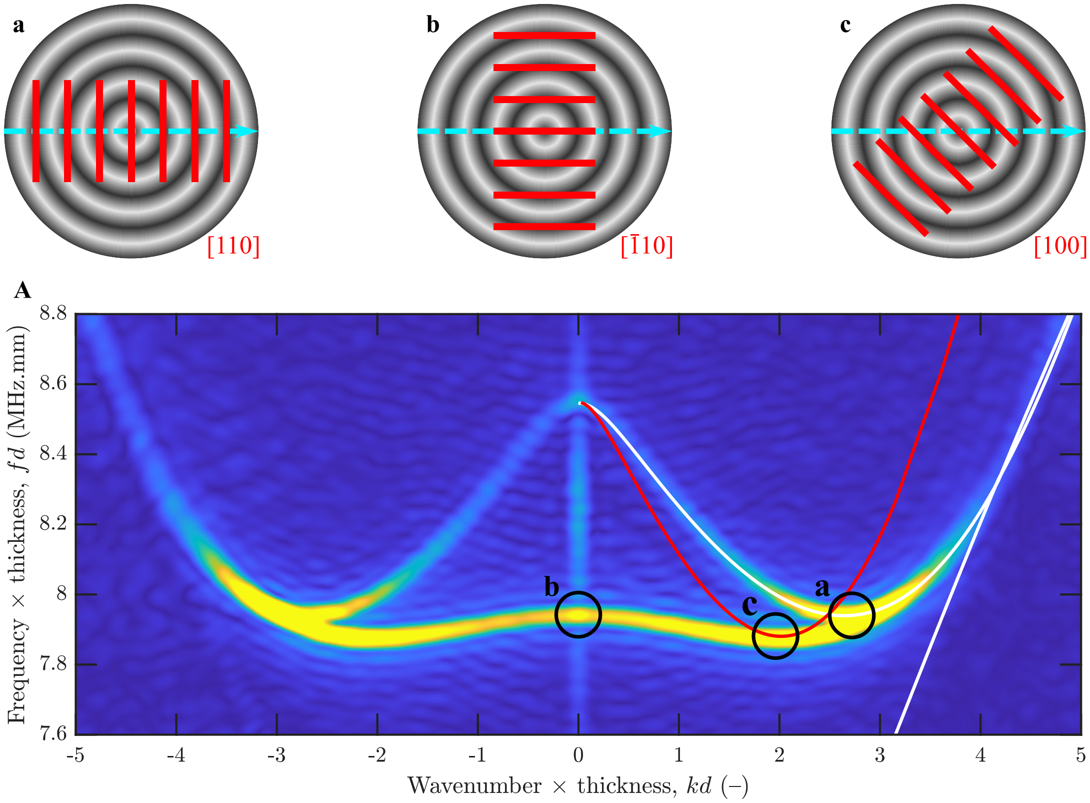

Supplementary Fig. 2: **Pseudo cut-off frequency and ZGV resonance splitting.** (A) Dispersion spectrum generated from a LUS measurement on bulk silicon along [1 1 0]. The laser excitation with a point source on the sample's surface triggers modes in all directions leading to a splitting of the measured modes in the dispersion spectrum. (a) The ZGV resonance at 7.94 MHz mm is assigned to the measurement direction [110]. (b) In contrast, the additional pseudo cut-off frequency at the same frequency can be assigned to the ZGV resonance along [$\bar{1}$ 1 0]. As it is perpendicular to the scanning direction, the wavenumber k appears to be zero. (c) The lower ZGV resonance at 7.88 MHz mm is linked to the [100] propagation direction and represents a signature of the material's anisotropy. The wavenumber is shifted by a factor of $\sqrt{2}$, as the angle between the wavefront and the measured direction is $\pi/4$.

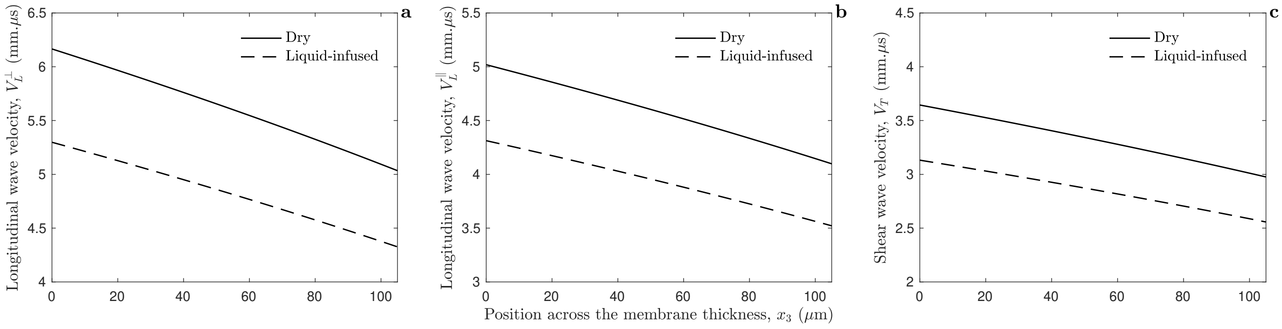

Supplementary Fig. 3: **Bulk wave velocity profiles across the membrane's thickness.** Bulk wave velocity profiles across the membrane's thickness, which result from the model parameterisation in terms of linearly varying stiffness-to-weight ratios: (a) Transverse longitudinal bulk wave velocity, $V_L^{\perp}$, (b) Axial longitudinal bulk wave velocity, $V_L^{\parallel}$, and (c) Shear bulk wave velocity, V_T.
