# Supplementary material for: Laser-excited elastic guided waves reveal the complex mechanics of nanoporous silicon
Source: Nat Commun. 2021 Jun 14;12:3597. doi: 10.1038/s41467-021-23398-0 (PMC8203614; doi:10.1038/s41467-021-23398-0)
Supplement: Supplementary file 3 — Description of Additional Supplementary Files [file 41467_2021_23398_MOESM3_ESM.pdf]

Description of Additional Supplementary Files:

Legend of Video - Supplementary Movie 1: „Visualisation of laser-excited guided waves. Side-view on the temporal evolution of elastic guided waves at a porous silicon surface. The movie has been constructed from a spatio-temporal laser-ultrasonics measurement.”
